# Perpendicularly oriented sub-10-nm block copolymer lamellae by atmospheric thermal annealing for one minute

**DOI:** 10.1038/srep19481

**Published:** 2016-01-19

**Authors:** Takehiro Seshimo, Rina Maeda, Rin Odashima, Yutaka Takenaka, Daisuke Kawana, Katsumi Ohmori, Teruaki Hayakawa

**Affiliations:** 1 Department of Organic and Polymeric Materials, Tokyo Institute of Technology, 2-12-1-S8-36 O-okayama, Meguro-ku, Tokyo 152-8552, Japan; 2 Tokyo Ohka Kogyo Co., Ltd, 1590 Tabata, Samukawa-machi, Koza-Gun, Kanagawa 253-0114, Japan; 3 Precursory Research for Embryonic Science and Technology (PREST), Japan Science and Technology Agency (JST), 4-1-8 Honcho, Kawaguchi, Saitama 332-0012, Japan

## Abstract

The directed self-assembly (DSA) of block co-polymers (BCPs) can realize next-generation lithography for semiconductors and a variety of soft materials. It is imperative to simultaneously achieve many requirements such as a high resolution, orientation control of micro-domains, etch selectivity, rapid and mild annealing, a low cost, and compatibility with manufacturing for developing suitable BCPs. Here, we describe a new design for modified polysiloxane-based BCPs targeted for sub-10-nm-wide lines, which are able to form perpendicularly oriented lamellar structures in thin films. The hydroxyl groups in the side chains introduced in the polysiloxane block provide a good balance with the polystyrene surface free energy, thereby leading to the perpendicular orientation. Moreover, this orientation can be completed in only one minute at 130 °C in an air atmosphere. Oxygen plasma etching for the thin films results in the achievement of a line width of 8.5 nm.

Nanostructure fabrication technologies have been developed to achieve a low cost, high resolution, and high throughput. One of the major technologies for soft materials is photolithography. However, current ArF immersion photolithography is now facing a resolution limit. Therefore, new lithography techniques are being developed to achieve a resolution less than 30 nm. One of these remarkable technologies is directed self-assembly (DSA) using self-assembled block co-polymers (BCPs) because it can form repeated features at such a scale^1^,^2^. To develop DSA techniques, many studies^3^,^4^,^5^,^6^,^7^,^8^ have been carried out using poly(styrene-*b*-methyl methacrylate) (PS-*b*-PMMA) because PS-*b*-PMMA can be perpendicularly oriented on a suitable substrate called a neutral layer after thermal annealing. This phenomenon is due to the similarity of the interfacial energies of the PS and PMMA blocks to the air interface^9^. However, the minimum pattern dimension L_0_ which can be achieved by self-assembly of PS-*b*-PMMA is approximately 24 nm, which corresponds to 12-nm-wide lines. The pattern dimension L_0_ is determined by both the interaction parameter χ and the degree of polymerization *N* because L_0_ scales as L_0_ ~ α*N*^2/3^χ^1/6^ in the strong-segregation limit (α is the characteristic segment length)^1^. Furthermore, the etch selectivity between PS and PMMA is not sufficiently high^10^. Therefore, new types of BCPs are required for next-generation materials. One candidate BCP is a Si-containing BCP such as poly(styrene-*b*-dimethylsiloxane) (PS-*b*-PDMS)^11^,^12^,^13^,^14^, poly(dimethylsiloxane-*b*-methyl methacrylate) (PDMS-*b*-PMMA)^15^, polyhedral oligomeric silsesquioxane (POSS) containing BCP^16^, poly(trimethylsilylstyrene-*b*-D,L-lactide)^17^, and poly(lactide-*b*-dimethylsiloxane-*b*-lactide)^18^. These BCPs have a higher value of χ than PS-*b*-PMMA and can therefore form sub-10-nm features and have a much higher etch selectivity because of the high etch resistance of the Si-containing block. Moreover, some of these BCPs can form high-resolution perpendicular domains after solvent annealing. Unfortunately, it is difficult to obtain a perpendicular pattern orientation for these Si-containing BCPs by thermal annealing, which is a more industrial-friendly method, even if a suitable neutral layer is applied to a substrate. This is because the Si-containing polymer block has lower surface free energy (SFE) and tends to segregate at the top surface of the BCP thin film. One solution for achieving a perpendicular orientation by thermal annealing includes topcoat approaches^19^,^20^. The process has been developed remarkably in recent year. Various kinds of orientation control methods have been reported in several Si-containing BCPs, which can form sub-10 nm features by thermal annealing^21^,^22^,^23^. Recently, Aissou *et al*. reported that a polycarbosilane-based BCP can be formed with sub-10-nm features by thermal annealing with a guide pattern^24^. However, few BCPs have achieved high-resolution perpendicular domains by thermal annealing without topcoat process up to now. To achieve a high resolution and perpendicular orientation by thermal annealing, BCP design should be very important. To form perpendicularly oriented features, the SFE of both block segments would be similar to the air interfaces. The classical high-χ BCPs which have only increased repulsion between the block segments should be formed with a parallel orientation because there is a difference in the SFEs between the two block segments.

In this work, we designed new BCPs to achieve a high resolution and perpendicular orientation by thermal annealing. We focused the compatibility between the SFEs of the inter-block and intra-block segments. Classical high-χ BCPs are composed of a hydrophobic block which has a lower SFE and a hydrophilic block which has a higher SFE. In our BCP design, the hydrophobic block has more hydrophilic end groups than the other hydrophilic blocks to balance the SFE between the two blocks. In this design, PS-*b*-PDMS was selected as a base BCP, and a hydroxyl group was used as a more hydrophilic end group. Because the hydroxyl group is expected to act as a repulsive group for the polystyrene block, the polysiloxane block exhibits a high etch resistance for oxygen plasma etching. Methyl vinyl siloxane was selected as the precursor of the modified polysiloxane block because the vinyl group can be introduced to functional side chain by the ene-thiol reaction or hydrosilylation. The hydrosilylation reaction requires a metal catalyst^25^. On the other hand, the ene-thiol reaction does not require a metal catalyst. Sometimes, the remaining metal components result in defects during nanofabrication process. Therefore, the ene-thiol reaction was selected to introduce a functional group to the side chain. In addition, the glass transition temperature of the siloxane main chain is very low. Therefore, a short time and low-temperature thermal annealing conditions are expected to promote the self-assembly of the BCP because of the high mobility of the BCP. Of course, a high etch selectivity is also expected because of the high etch resistance of the siloxane block. We have also examined the effect of the side-chain structure for micro-phase separation. Moreover, we have also attempted to find the process conditions for forming perpendicular domains by rapid and mild thermal annealing.

## Results and Discussion

**Figure 1:**
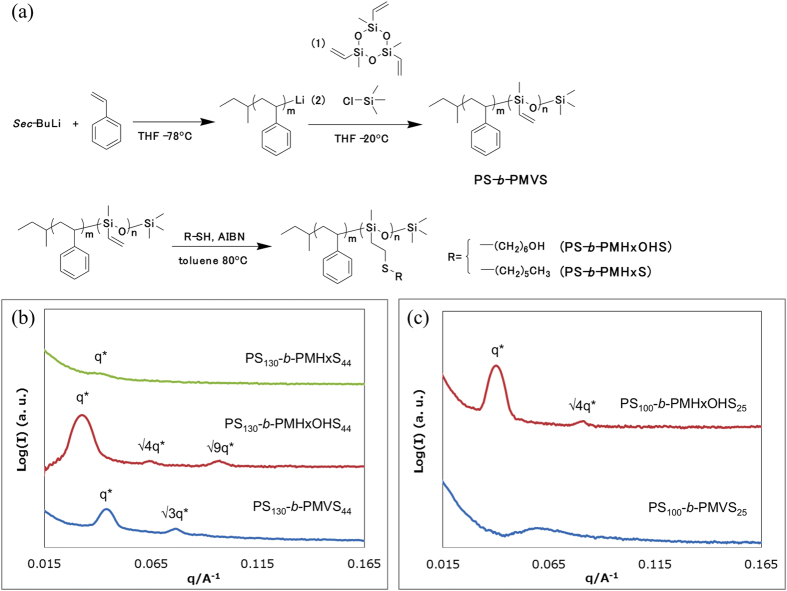
(**a**) Synthesis scheme for PS-*b*-PMVS and vinyl-group-modified BCPs. SAXS profiles of (**b**) the BCPs in Group H in Table 1 collected after thermal annealing at 190 °C for 3 h and (**c**) the BCPs in Group L in Table 1 collected after thermal annealing at 130 °C for 3 h.

All of the poly(styrene-*b*-methyl vinyl siloxane) (PS-*b*-PMVS) BCPs, which are precursor BCPs with different molecular weights and unit ratios, were synthesized by anionic polymerization of styrene and 1,3,5-trimethyl-1,3,5-trivinyl cyclotrisiloxane. After reaction, the vinyl group on the siloxane unit was modified with two types of thiol reagents to provide poly(styrene-*b*-substituted siloxane)s, namely, PS-*b*-PMHxS and PS-*b*-PMHxOHS (the M, Hx and HxOH indicate methyl, hexyl and hexanol, respectively). The synthesis scheme is shown in Fig. 1(a). The details of the synthesis procedure and characterization results of the BCPs are described in the Supplementary Information. The BCPs used in this study are summarized in Table 1. The BCPs in Group L have a lower molecular weight, and those in Group H have a higher molecular weight.

The bulk morphologies of the BCPs were characterized by small-angle X-ray scattering (SAXS). For the BCPs in Group H, PS_130_-*b*-PMHxS_44_ (the subscripts indicate the number of repeat monomer units), which was reacted with 1-melcapt hexane, exhibited a single peak, which means no ordered structure was formed because the introduced alkyl side chain has less repulsion with the PS block, which should decrease χ. The other BCPs exhibited more than two peaks, which indicates the formation of higher-ordered structures in the case of a higher molecular weight [Fig. 1(b)]. On the other hand, for the BCPs in Group L, PS_100_-*b*-PMHxOHS_25_, which has a hydroxyl end group, exhibited more than two peaks [Fig. 1(c)]. These results revealed that the side-chain structure affects the micro-phase separation. The SAXS measurement results indicated that the domain spacing of PS_100_-*b*-PMHxOHS_25_ was 16 nm, and the morphology was lamellar.

**Figure 2:**
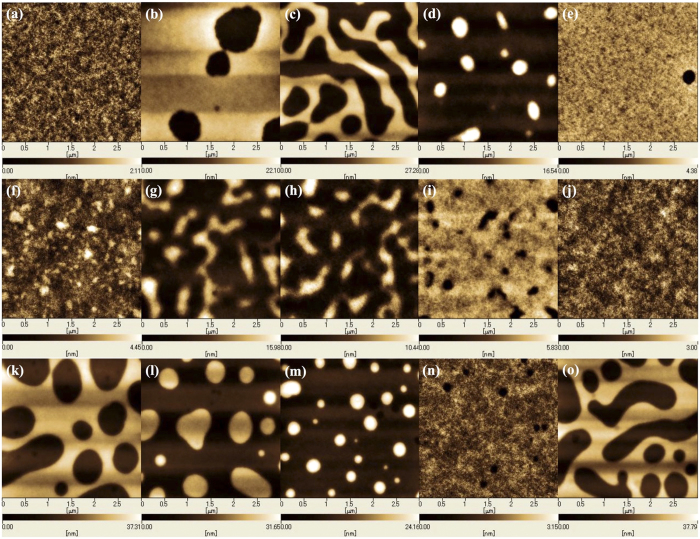
AFM height images of different thicknesses of PS_100_-*b*-PMHxOHS_25_ after annealing at 130 °C for 1 min (**a–e**) on PS-*r*-PMA, (**f–j**) on PMMA-*r*-PMA, and (**k–o**) on poly hydroxyethyl methacrylate (PHEMA). The PS_100_-*b*-PMHxOHS_25_ thicknesses were (**a**,**f**,**k**) 32 nm (=2.0L_0_); (**b**,**g**,**l**) 28 nm (=1.8L_0_); (**c**,**h**,**m**) 24 nm (=1.5L_0_); **(d,i**,**n**) 20 nm (=1.3L_0_); and (**e**,**j**,**o**) 16 nm (=1.0L_0_). Fingerprint patterns were observed for (**f**,**i**). No image was observed for the others.

A self-assembly study of the thin films for forming lamellar structures was carried out for PS_100_-*b*-PMHxOHS_25_ (the domain spacing is 16 nm by SAXS). We focused on the neutral layer, the film thicknesses of the BCPs, the annealing temperature, and the casting solvent. Firstly, the optimum neutral layer for the BCP was determined. Three types of non-Si-containing random copolymers (RCPs) were tested, as summarized in the Supplementary Information. The classical design of the neutral layer is an RCP made from the same monomers as the BCP with a cross-linkable or covalently bondable functional group^3^,^26^. However, the same design cannot be applied for Si-containing BCPs because the Si-containing neutral layer should have a high etch resistance during oxygen plasma etching, which is not ideal for pattern transfer to the substrate. The details of the experimental procedure are described in the Supplementary Information. As a result, poly(methacrylate-*r*-methacrylic acid) (PMMA-*r*-PMA) acts as a neutral layer for PS_100_-*b*-PMHxOHS_25_ [Fig. 2(b,g,l)] because terrace structures with half of the size of the bulk periodicity (0.5L_0_) in height were observed only on PMMA-*r*-PMA^27^. The cross-sectional profiles of Fig. 2(b,g,l) are shown in the Supplementary Information.

Secondly, the film-thickness dependency was also examined using the same RCPs. As a result, the film thicknesses of 1.0L_0_ or 2.0L_0_ on PMMA-*r*-PMA exhibited a fingerprint pattern, which means a perpendicular orientation should be achieved [Fig. 2(f,j)]. Some flat surfaces were observed when the film thickness was 1.0L_0_ or 2.0L_0_ on PS-*r*-PMA, but there were no patterns [Fig. 2(a,e)]. The surface of PS-*r*-PMA is PS preferential; therefore, a complete parallel lamellar structure was formed with a thickness of 1.0L_0_ or 2.0L_0_, and a PS block covered the BCP surface. The contact-angle measurements assumed that the surfaces consisted of a PS-block wetting layer because the surface conditions were almost equivalent to the surface of a PS homopolymer film. Of course, the surface consisting of a fingerprint pattern exhibited different surface conditions (see Supplementary Information).

**Figure 3:**
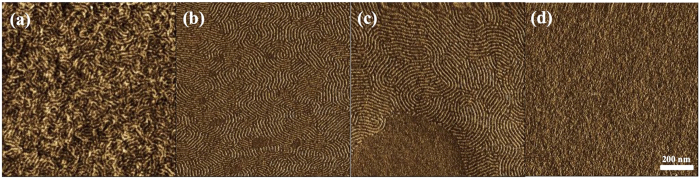
AFM phase images of a 32-nm-thick (=2.0L_0_) PS_100_-*b*-PMHxOHS_25_ film (**a**) before annealing, (**b**) after annealing at 130 °C for 1 min, (**c**) after annealing at 140 °C for 1 min, and (**d**) after annealing at 150 °C for 1 min with a PMMA-*r*-PMA film.

Thirdly, the effect of the annealing temperature for orientation control was examined using PS_100_-*b*-PMHxOHS_25_ on a PMMA-*r*-PMA film. In this experiment, annealing time is fixed to one minute from a view point of throughput. Under these conditions, a fingerprint pattern was observed before annealing; however, the correlation length was not long enough [Fig. 3(a)]. After annealing at 130 °C for 1 min, the correlation length became longer, and the pattern profile became better [Fig. 3(b)]. However, after annealing at 140 °C for 1 min, some terrace structures were observed [Fig. 3(c)]. Moreover, no fingerprint pattern was observed after annealing at 150 °C for 1 min [Fig. 3(d)]. According to these results, 130 °C for 1 min should be the best annealing conditions for PS_100_-*b*-PMHxOHS_25_. The same experiment to find the best annealing condition was also executed with PS_100_-*b*-PMHxOHS_25_ which had higher molecular weight. The results are shown in Figure S8 in Supplementary Information. The results indicate that PS_100_-*b*-PMHxOHS_25_ can also form perpendicularly oriented lamellar with PMMA-*r*-PMA. It notes that the suitable annealing temperature shifts to higher temperature around 150–160 °C. These annealing conditions are much milder than the annealing conditions for PS-*b*-PMMA, which are typically greater than 200 °C or over 30 min^28^. These conditions are attractive for achieving a short process time during manufacturing. The appropriate annealing conditions promote the formation of the micro-phase separation from the state before annealing. On the other hand, higher annealing conditions cause a drastic orientation transformation. Therefore, one block covered the BCP film surface and formed a terrace structure or PS wetting layer.

**Figure 4:**
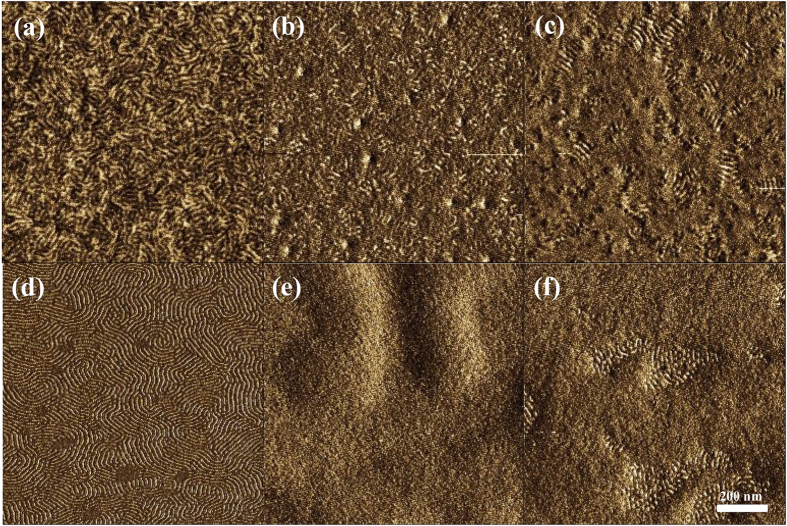
AFM phase images of a 32-nm-thick (=2.0L_0_) PS_100_-*b*-PMHxOHS_25_ film cast from different solvents (**a**–**c**) before annealing and (**d–f**) after annealing at 130 °C for 1 min. (**a,d**) Cast from a PGMEA solution. (**b,e**) Cast from a toluene solution. (**c,f**) Cast from a chloroform solution with a PMMA-*r*-PMA film.

Finally, the impact of the casting solvent was investigated. In this experiment, all experimental conditions, except the casting solvent, were the same. The BCP film cast from propylene glycol monomethyl ether acetate (PGMEA) exhibited a fingerprint-like pre-pattern [Fig. 4(a)]. On the other hand, the BCP films cast from toluene and chloroform exhibited no patterns [Fig. 4(b,c)]. After annealing at 130 °C for 1 min, only the films cast from PGMEA exhibited fingerprint patterns [Fig. 4(d–f)]. These results are caused by the difference in the BCP state before annealing. PGMEA has a higher boiling point; therefore, PGMEA slowly evaporated from the BCP film during spin coating. At this time, micro-domains should be assembled from the top surface of the BCP film, similar to solvent annealing^29^. Although PS_100_-*b*-PMHxOHS_25_ cannot form perpendicular lamellae after solvent annealing, the use of toluene and chloroform with PMMA-*r*-PMA can form perpendicular lamellae after solvent annealing using PGMEA. These results are shown in the Supplementary Information. Thus, PGMEA is a good solvent for realizing a perpendicular orientation for PS_100_-*b*-PMHxOHS_25_. Moreover, PGMEA is used as a common casting solvent in industry. In addition, a uniform thin film was observed when casting from PGMEA, and no de-wetting was observed during the thermal annealing process.

**Figure 5:**
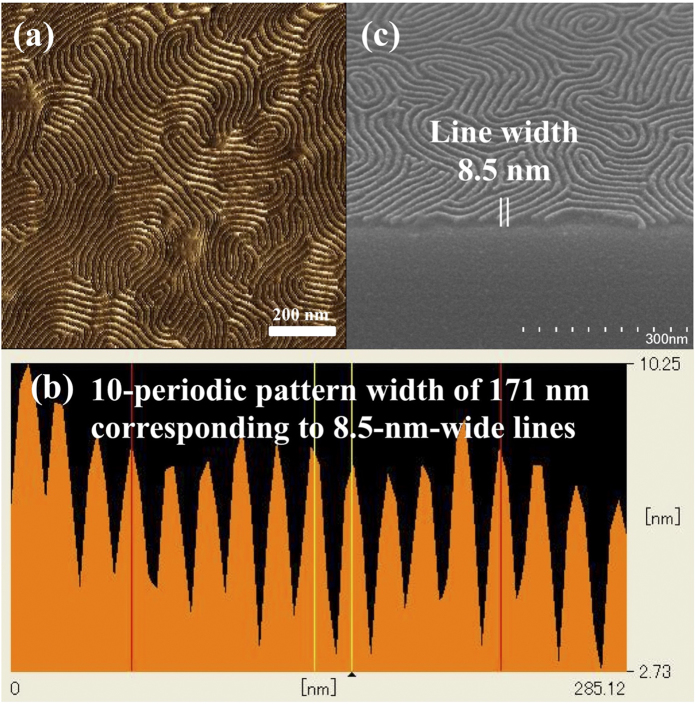
(**a**) AFM phase image of a 32-nm-thick (=2.0L_0_) PS_100_-*b*-PMHxOHS_25_ film after annealing at 130 °C for 1 min and O_2_-RIE for 90 s. The etching rate for PS is 0.25 nm/s under these conditions. (**b**) AFM height cross section of the image in (**a**). (**c**) SEM image of (**a**).

For DSA technology, the etching selectivity of the BCP is also important. Therefore, the BCP should consist of two blocks which have etching selectivity. Generally, polysiloxane exhibits a higher etch resistance to oxygen plasma etching than hydrocarbon polymers such as PS or PMMA^30^. In the case of PS_100_-*b*-PMHxOHS_25_, the PMHxOHS block has a higher etch resistance than the PS block. If the orientation control is not perfect, a dislocated PMHxOHS block might act as etch barrier to prevent pattern transfer to the substrate. Therefore, exact orientation control would be required. To achieve successful orientation control, the BCP characteristics and process conditions for the BCP are important. Moreover, many BCPs cannot form perpendicular domains by thermal annealing because of the difference in the SFEs of the two blocks, especially the high-χ BCP, to achieve smaller feature sizes than those of PS-*b*-PMMA, even if a suitable neutral layer is applied. An atomic force microscopy (AFM) image and a cross-sectional scanning electron microscopy (SEM) image of a 32-nm-thick (=2.0L_0_) PS_100_-*b*-PMHxOHS_25_ film after annealing at 130 °C for 1 min with PMMA-*r*-PMA and oxygen-plasma reactive-ion etching (O_2_-RIE) for 90 s are shown in Fig. 5. The AFM images exhibited a similar pattern profile before etching [Fig. 5(a)]. Furthermore, the cross-sectional profile of the AFM measurements also supported the perpendicular orientation because a highly ordered periodic pattern was observed. The results indicated 8.5-nm-wide lines were generated by O_2_-RIE [Fig. 5(b)]. Moreover, the SEM image also evidenced the formation of perpendicular domains [Fig. 5(c)]. These results indicated that these patterns were formed not only near the surface but also inside the film, which means a perpendicular orientation should be achieved after annealing at 130 °C for 1 min. From the homopolymer etching rate measurement, PS is removed faster during etching, and the etch rate of PS is 0.25 nm/s under these conditions.

Ideal BCP materials for DSA should have a high etching selectivity, the ability to form perpendicular domains on the substrate by thermal annealing under mild condition, and a high resolution. In particular, a BCP which can form a domain spacing less than 24 nm is desirable because PS-*b*-PMMA cannot form such small features. The classical approach for achieving a smaller feature is to increase the repulsion, e.g. by introducing Si-containing blocks. However, the polymer design also causes an orientation control problem, which results in a horizontal orientation because of the large difference in the SFEs between the two blocks. To overcome this problem, we designed a new BCP and optimized the process conditions to achieve these characteristics. PS_100_-*b*-PMHxOHS_25_ can be formed with perpendicular lamellae with an 8.5-nm-wide line on PMMA-*r*-PMA after annealing at 130 °C for 1 min with the appropriate conditions. Moreover, the etching process also successfully removed PS blocks with O_2_-RIE. The key factor for achieving these characteristics is the polymer design. The main chain of PMHxOHS consisting of polysiloxane has greater hydrophobicity than the PS block. On the other hand, the hydroxyl groups on the PMHxOHS side chain exhibit hydrophilicity. Therefore, the PMHxOHS block has properties similar to an amphipathic molecule. In the design, the hydroxyl groups prevent the main chain from forming a wetting layer. Further, both the hydroxyl groups and polysiloxane main chains are repulsed by the PS block, which enables smaller-feature micro-phase separation. On the other hand, the side chain of PMHxS has an alkyl chain, which has a low repulsion with the PS block. Therefore, PS_130_-*b*-PMHxS_44_ cannot form smaller micro-phase-separated features. This new type of polymer design could be expanded to achieve smaller-feature formation and orientation control simultaneously, which is very useful for industrial nanofabrication and attractive to basic polymer science for academic study.

## Methods

### Preparation of poly(styrene-*b*-methyl vinyl siloxane) (PS-*b*-PMVS)

Styrene was distilled over dibutylmagnesium. 1,3,5-trimethyl-1,3,5-trivinyl cyclotrisiloxane (MVCtS) was distilled over CaH_2_. Tri-methyl silyl chloride (TMSiCl) was degassed under Ar. Azobisisobutyronitrile (AIBN) was recrystallized with methanol. All monomers for neutral layer were distilled under vacuum. Other reagents and solvents were used as received. *Sec*-butyl lithium and styrene were stirred in THF at −78 °C for 30 min under Ar atmosphere. MVCtS was added into the reactor. After 10 min, the reaction temperature was increase to −20 °C. After 48 h, TMSiCl was added into the reactor. The resulting polymer was precipitated in methanol.

### Preparation of thiol modified BCPs

PS-*b*-PMVS and AIBN and thiol reagent (6-mercapt 1-hexanol, 1-mercapt hexane) were stirred in toluene at 80 °C for 60 min under N_2_ atmosphere. The resulting polymer was precipitated in hexane and methanol. The ^1^H-NMR spectra are shown in Supplementary Information.

### Preparation of RCPs

Every monomers and AIBN were stirred in THF at 80 °C for 18 h under N_2_ atmosphere. The resulting polymer was precipitated in methanol.

### Characterization in bulk

The bulk samples were prepared from chloroform solution. After evaporating the solvent, the samples were annealed under vacuum at each temperature. The bulk morphologies of the BCPs were characterized by small-angle X-ray scattering (SAXS). The SAXS measurement was performed using a Bruker NanoSTAR with a 2D-PSPC detector.

### Characterization in thin film

RCPs were spin-coated onto a Si substrate from a solution (1.0 wt % in propylene glycol methyl ether acetate (PGMEA) for PS-*r*-PMA and PMMA-*r*-PMA, or methanol for PHEMA) at 3000 rpm and subsequently baked at 200 °C for 1 min on a hot plate. Then rinse was executed using each casting solvent to remove unreacted polymer. BCPs were spin-coated onto the rinsed substrate from a solution (1.0 ~ 1.5 wt % in PGMEA). The spin-coated thin films were annealed on the hot plate.

Oxygen plasma etching was carried out in vacuum chamber. Oxygen flow rate was 40 sccm and applied voltage was 20 W.

Thin films were characterized by scanning electron microscopy (SEM) (Hitachi SU9000) and atomic force microscopy (AFM) (SII SPA400). AFM was utilized in tapping mode, with SI-DF20 (Hitachi High-Tech Science) to measure the height profiles and the phase images of the BCP thin films. Before SEM observation, the sample was coated with Pt using spattering.

### Solvent annealing

PMMA-*r*-PMA was spin-coated onto a Si substrate from a solution (1.0 wt % in PGMEA) at 3000 rpm and subsequently baked at 200 °C for 1 min on a hot plate. Then rinse was executed using PGMEA to remove unreacted polymer. PS_100_-*b*-PMHxOHS_25_ was spin-coated onto the rinsed substrate from a solution (1.5 wt % in PGMEA). The wafer was put in a capped vessel with 10 ml each solvent at room temperature and atmospheric pressure.

## Additional Information

**How to cite this article**: Seshimo, T. *et al*. Perpendicularly oriented sub-10-nm block copolymer lamellae by atmospheric thermal annealing for one minute. *Sci. Rep.* **6**, 19481; doi: 10.1038/srep19481 (2016).

## Supplementary Material

Supplementary Information

## Figures and Tables

**Table 1:** Characterizations of PS-*b*-PMVSs and PS-*b*-PMHxOHSs.

**Group**	**Sample**	***M***_**n**_ **(g/mol)**	**PDI**^a^	wt% ofPS^b^	**Morphology**	***d*****-spacing (nm)**^c^	**R**
H	PS_130_-*b*-PMVS_44_	17,500	1.06	78	cylinder	14	-
L	PS_100_-*b*-PMVS_25_	12,700	1.06	83	disordered	-	-
H	PS_130_-*b*-PMHxOHS_44_	20,500	1.13	57	lamellar	19	-(CH_2_)_6_OH
L	PS_100_-*b*-PMHxOHS_25_	14,500	1.15	65	lamellar	16	-(CH_2_)_6_OH
H	PS_130_-*b*-PMHxS_44_	20,000	1.06	60	disordered	-	-(CH_2_)_5_CH_3_

^a^Polydispersity index (PDI) was determined by size exclusion chromatography.

^b^PS weight fraction was determined by ^1^H-NMR spectra.

^c^Morphology and domain spacing were evaluated by small angle X-ray scattering (SAXS) profile and transmission electron micrograph.
