# Supplementary material for: Perpendicularly oriented sub-10-nm block copolymer lamellae by atmospheric thermal annealing for one minute
Source: Sci Rep. 2016 Jan 19;6:19481. doi: 10.1038/srep19481 (PMC4726028; doi:10.1038/srep19481)
Supplement: Supplementary Information [file srep19481-s1.pdf]

Supplementary Information

Perpendicularly oriented sub-10-nm block copolymer lamellae by atmospheric thermal annealing for one minute

Takehiro Seshimo[1, 2], Rina Maeda[1], Rin Odashima[1], Yutaka Takenaka[1], Daisuke Kawana[2], Katsumi Ohmori[2], Teruaki Hayakawa[1, 3]*

[1]Department of Organic and Polymeric Materials, Tokyo Institute of Technology, 2-12-1-S8-36 O-okayama, Meguro-ku, Tokyo 152-8552, Japan

[2]Tokyo Ohka Kogyo Co., Ltd, 1590 Tabata, Samukawa-machi, Koza-Gun, Kanagawa 253-0114, Japan

[3]Precursory Research for Embryonic Science and Technology (PREST), Japan Science and Technology Agency (JST), 4-1-8 Honcho, Kawaguchi, Saitama 332-0012, Japan

*hayakawa.t.ac@m.titech.ac.jp

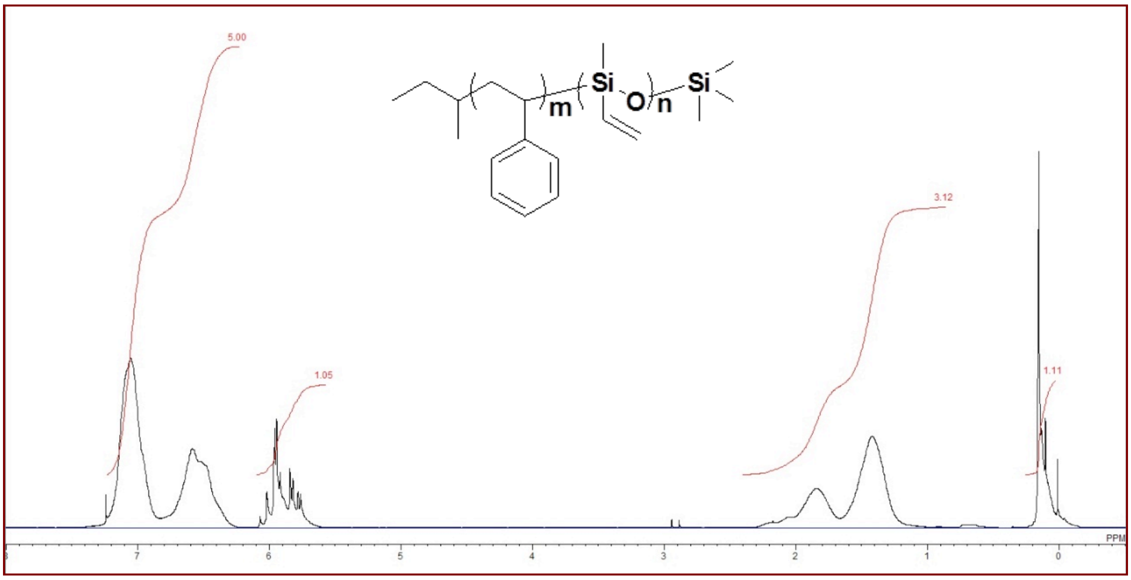

Figure S1. ^{1}H-NMR spectra for PS$_{130}$-*b*-PMVS$_{44}$.

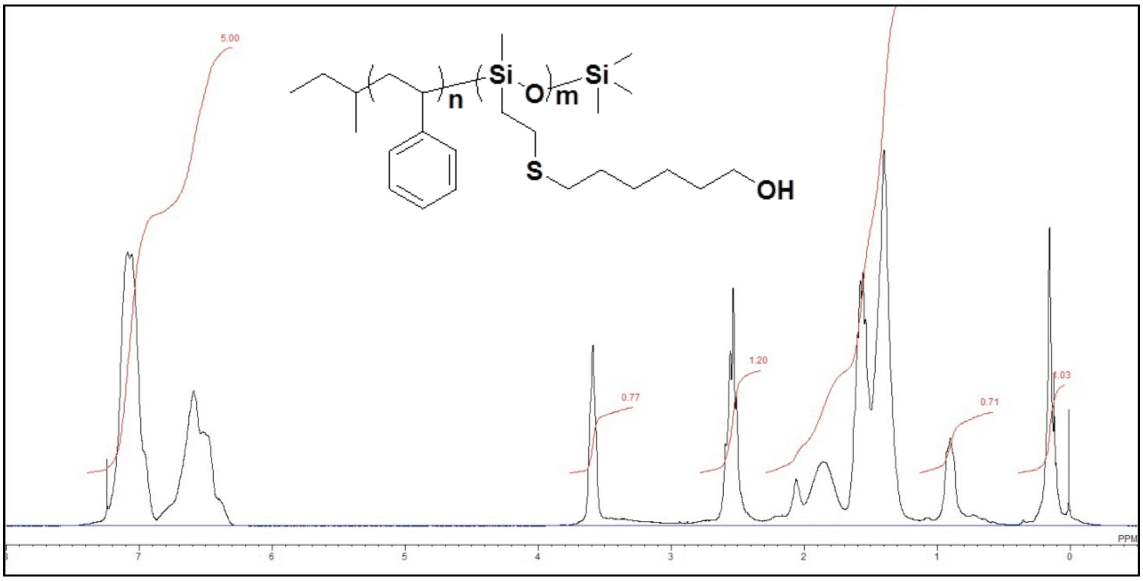

Figure S2. ^{1}H-NMR spectra for PS$_{112}$-*b*-PMHxOHS$_{40}$.

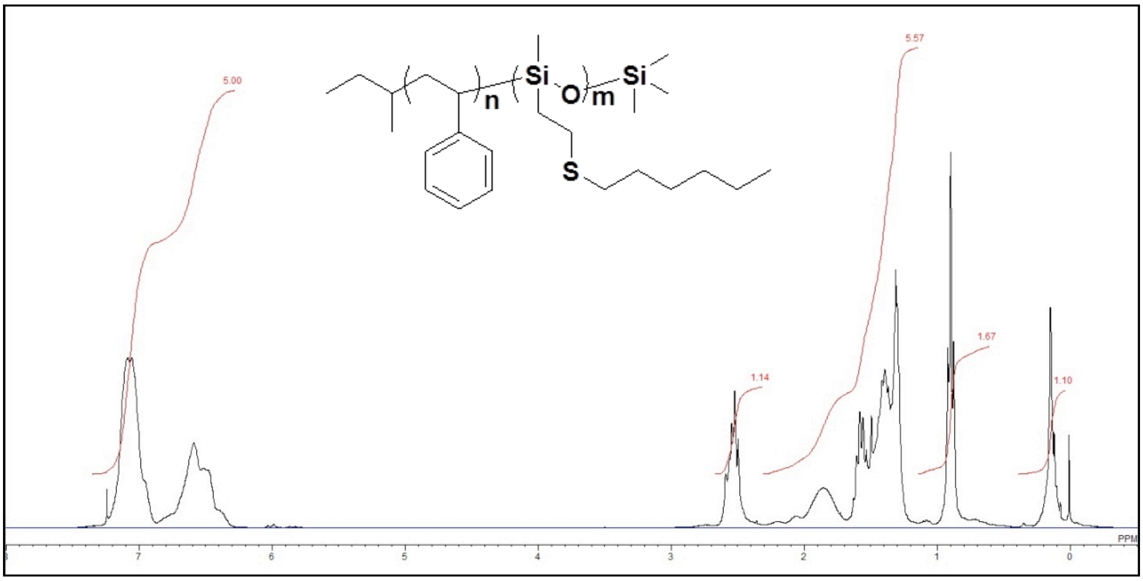

Figure S3. ^{1}H-NMR spectra for PS_{115}-*b*-$PMHxS_{39}$.

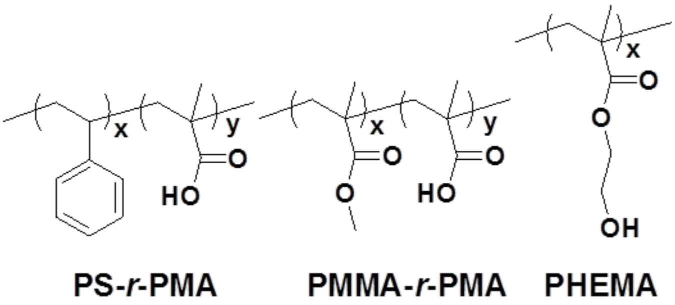

Figure S4. Chemical structure of the RCPs used in this study

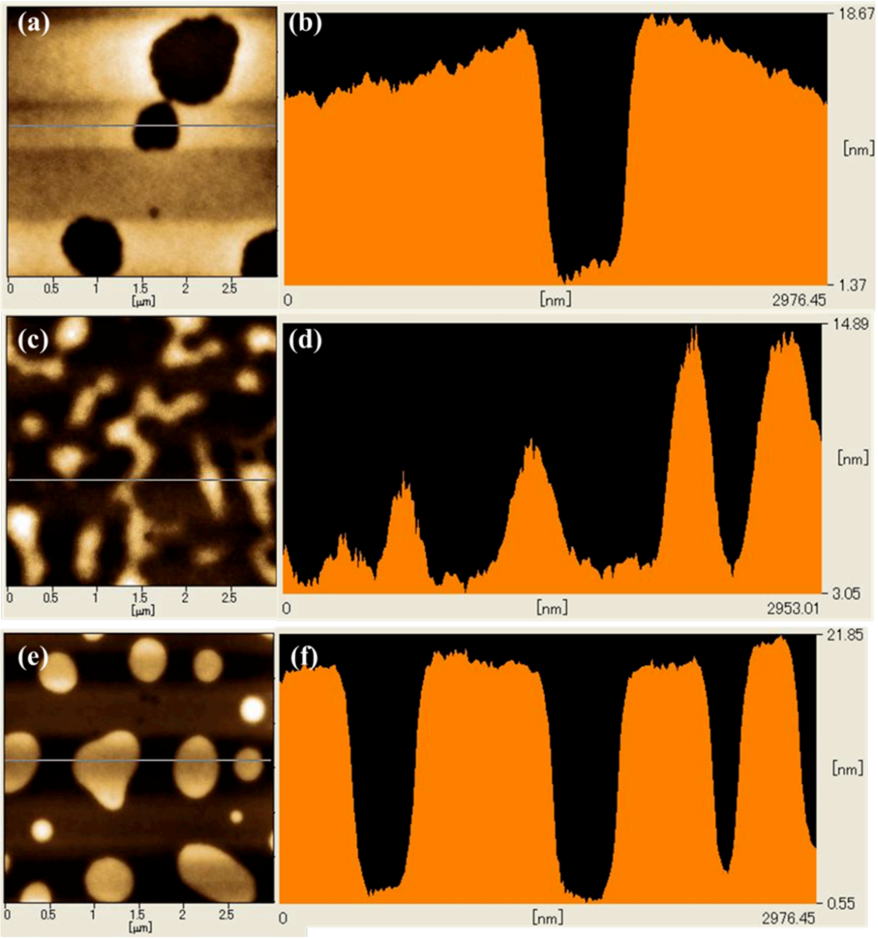

Figure S5. AFM height images and cross section profile of different thick PS_{90}-*b*-$PMHxOHS_{23}$ after annealed at 130 °C for 1 min (a)–(b) on PS-*r*-PMA (c)–(d) on PMMA-*r*-PMA (e)–(f) on PHEMA. The cross section area is shown by the bar in height image.

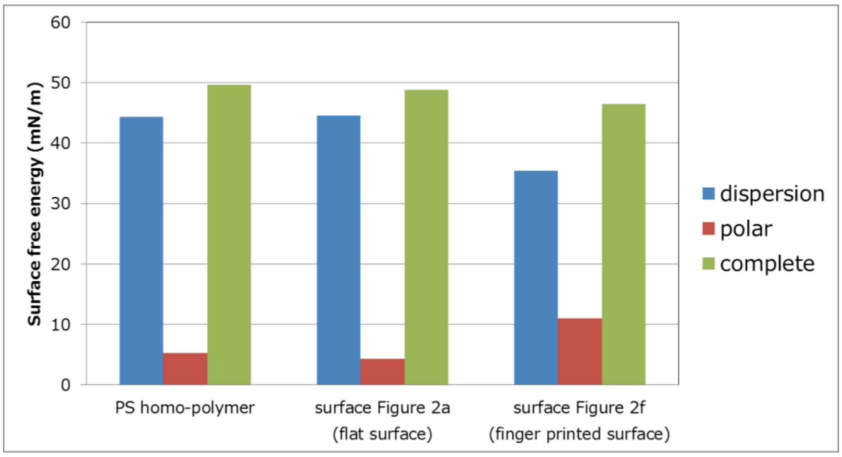

Figure S6. Calculated surface free energy of PS homo-polymer surface and annealed PS_{90}-*b*-$PMHxOHS_{23}$ thin film surface on different RCP film. Static contact angles were measured with H_2O and diiodomethane. Surface free energy of each surface was calculated using the dispersion and polar value of liquids with Wu method equations.

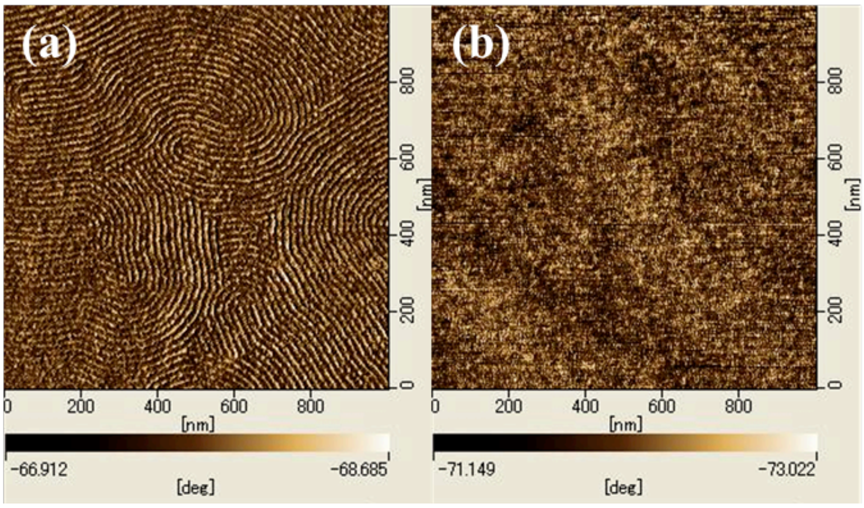

Figure S7. AFM phase images of PS_{90}-*b*-$PMHxOHS_{23}$ after solvent annealed using (a) PGMEA (b) toluene for 2 h with PMMA-*r*-PMA.

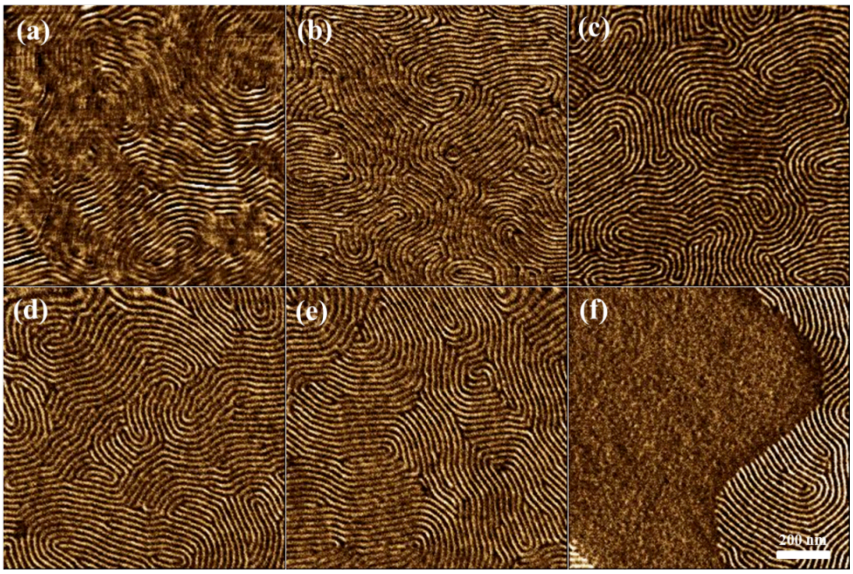

Figure S8. AFM phase images of a 19-nm-thick (=1.0L_0) PS_{112}-*b*-$PMHxOHS_{40}$ film (a) before annealing, (b) after annealing at 130 °C for 1 min, (c) after annealing at 140 °C for 1 min, (d) after annealing at 150 °C for 1 min, (e) after annealing at 160 °C for 1 min, and (f) after annealing at 170 °C for 1 min with a PMMA-*r*-PMA film.

Table S1. Properties of the BCPs investigated in this study

Sample	M_n (g/mol)	Dispersity	Unit ratio	Water C. A. (°)
PS-*r*-PMA	17,300	1.71	95 : 5	84
PMMA-*r*-PMA	31,100	1.50	95 : 5	63
PHEMA	41,000	1.76	100	46
